# Somatic variants of potential clinical significance in the tumors of *BRCA* phenocopies

**DOI:** 10.1186/s13053-019-0117-5

**Published:** 2019-07-16

**Authors:** Lela Buckingham, Rachel Mitchell, Mark Maienschein-Cline, Stefan Green, Vincent Hong Hu, Melody Cobleigh, Jacob Rotmensch, Kelly Burgess, Lydia Usha

**Affiliations:** 1 Department of Pathology, Rush University Medical Center, Chicago, IL USA; 2 Rush Cancer Institute, Rush University Medical Center, Chicago, IL USA; 3 Tennessee Oncology, Shelbyville, TN USA; 4 University of Illinois at Chicago Research Resources Center, Chicago, IL USA

**Keywords:** Phenocopy, HBOC, NGS, Somatic mutations

## Abstract

**Background:**

*BRCA* phenocopies are individuals with the same phenotype (i.e. cancer consistent with Hereditary Breast and Ovarian Cancer syndrome = HBOC) as their affected relatives, but not the same genotype as assessed by blood germline testing (i.e. they do not carry a germline *BRCA1* or *BRCA2* mutation). There is some evidence of increased risk for HBOC-related cancers in relatives of germline variant carriers even though they themselves test negative for the familial variant (*BRCA* non-carriers). At this time, *BRCA* phenocopies are recommended to undergo the same cancer surveillance as individuals in the general population. This raises the question of whether the increased cancer risk in *BRCA* non-carriers is due to alterations (germline, somatic or epigenetic) in other cancer-associated genes which were not analyzed during *BRCA* analysis.

**Methods:**

To assess the nature and potential clinical significance of somatic variants in *BRCA* phenocopy tumors, DNA from *BRCA* non-carrier tumor tissue was analyzed using next generation sequencing of 572 cancer genes. Tumor diagnoses of the 11 subjects included breast, ovarian, endometrial and primary peritoneal carcinoma. Variants were called using FreeBayes genetic variant detector. Variants were annotated for effect on protein sequence, predicted function, and frequency in different populations from the 1000 genomes project, and presence in variant databases COSMIC and ClinVar using Annovar.

**Results:**

None of the familial *BRCA1/2* mutations were found in the tumor samples tested. The most frequently occurring somatic gene variants were *ROS1*(6/11 cases) and *NUP98* (5/11 cases). *BRCA2* somatic variants were found in 2/6 *BRCA1* phenocopies, but 0/5 *BRCA2* phenocopies. Variants of uncertain significance were found in other DNA repair genes (*ERCC1, ERCC3, ERCC4, FANCD2, PALB2*), one mismatch repair gene (*PMS2*), a DNA demethylation enzyme (*TET2*), and two histone modifiers (*EZH2, SUZ12*).

**Conclusions:**

Although limited by a small sample size, these results support a role of selected somatic variants and epigenetic mechanisms in the development of tumors in *BRCA* phenocopies.

## Introduction

Cancer predisposition in hereditary breast and ovarian cancer (HBOC) syndrome is caused by pathogenic germline variants in the *BRCA1/2* genes (*germline BRCA* variants) and is inherited in an autosomal dominant pattern. The lifetime risk of breast cancer in female *germline BRCA* variant carriers is up to 85%; the lifetime risk of ovarian cancer is up to 50% [2, 6, 8, 11, 33, 34, 36, 38] Genetic alterations in the *BRCA1/2* genes cause over 90% of cases of HBOC [6, 33, 34, 36]. The genetic test recommended for patients suspected of carrying a *germline BRCA* variant involves sequencing of their *BRCA1* and *BRCA2* genes with deletion/duplication analyses, most commonly in blood and less frequently in saliva. A relative of a *germline BRCA* variant carrier who has tested negative for the known familial alteration is deemed to have normal (wild-type) germline *BRCA1* and *BRCA2* genes and is sometimes called a *BRCA* non-carrier.

There are conflicting reports on the relative risk ratio (RR) of breast and ovarian cancers in *BRCA* non-carriers when there is a known familial *BRCA* genetic alteration. Some authors argue that their cancer risk is the same as in the general population [24, 42], some conclude that their risk is the same as high risk families without identified germline *BRCA* pathogenic variants [20], but, overall, most authors agree that the risk is increased with a breast cancer RR of up to 5.1 [14, 27, 32, 35]. The studies are difficult to compare due to differences in methodology and patient populations [17] as well as different prognoses of breast cancers with *BRCA1* vs. *BRCA2* alterations [12, 25]. Germline *BRCA* non-carriers that develop breast or ovarian cancers are referred to as “*BRCA* phenocopies,” meaning that they have the same phenotype (affected by cancer) as germline *BRCA* carriers, but do not have the same genotype (the known *BRCA* alteration as shown by germline genetic testing).

Explanations offered for HBOC malignancies in *BRCA* phenocopies include sporadic cancer related to familial lifestyle and/or environmental factors or pathogenic variants in other, possibly not yet identified, genes that cause HBOC. All of these explanations assume that cancers in *BRCA* non-carriers are not related to the familial *BRCA* variant. The risk of *BRCA* non-carriers developing an HBOC cancer is clinically important because it determines their cancer surveillance and prevention recommendations [16].

Undetectable germline variants in blood might also be due to revertant mosaicism where a rare spontaneous correction of a pathogenic variant might occur in *BRCA* pedigrees and result in false-negative testing for the familial variant. However, a study by Azzollini et al. [4] did not find the familial variants in tumor samples, blood leukocytes, buccal mucosa nor urine of *BRCA* phenocopies. We previously explored natural chimerism as an alternative explanation for *BRCA* phenocopies [28]. We hypothesized that breast and ovarian cancer can still be caused by familial *BRCA* variants, but transmitted in an alternative, non-mendelian fashion (e.g., through maternal-fetal or tetragametic chimerism) so that the altered genes are present in chimeric tissues rather than in blood. Since *BRCA* mutant cells are much more likely to give rise to cancer than non-mutants cells, in a chimeric organism, the tumor would be *BRCA*-mutant. We analyzed tumor tissue in *BRCA* phenocopies for the known familial variant using targeted PCR and qPCR methods [28]. In our cohort of 11 cases, we did not find the familial alteration in the tumor tissues. In the current study, we analyzed the tumor samples from the same cohort of patients. We used next generation sequencing (NGS) to investigate the possibility of other (somatic and/or germline) gene variants driving the cancer phenotype and the possibility of *BRCA1/2* epigenetic silencing in the context of familial cancer predisposition.

## Methods

### Patients, clinical assessment, and germline genetic testing

Patients for this study were selected based on an HBOC cancer phenotype in the absence of a known familial *BRCA* mutation found in a first-degree relative. With approval by the Rush University Medical Center Institutional Review Board, each subject signed an informed consent form and tumor specimens were obtained from the Department of Pathology, Rush University Medical Center (Chicago, IL) and Pathology Departments of other institutions where participants had their cancer surgery. Cancer diagnoses were obtained from pathology reports and histologic evaluation. Clinical data were established from chart review and self-reported history forms. Patients were eligible if they were affected by cancer but had previously tested negative for a known familial pathogenic variant. Breast cancer patients under 45 (invasive or non-invasive), women with ovarian, fallopian tube, or primary peritoneal cancer at any age, endometrial cancer, patients with male breast cancer or pancreatic cancer were considered eligible for this study. Eleven cases that met these criteria were found. Familial mutations included *BRCA1* c.186_187delAG (p.L22_E23LVfs; 2 patients), c.1793delA (p.G559Vfs), c.17 + 3A > G, c.2841A > T (p.K947 N), c.3109_3110insAA (p.K1037 fs), c.5215G > A (p.D1739N), c.8107G > A, and *BRCA2* c.6794_6975 insA, c.5645C > A (p.S882*) and c.6174delT (p.F2058Lfs).

Four patients underwent expanded commercial germline genetic testing in addition to the *BRCA1/2* testing. Patients 1 and 9 underwent gene panel testing that included 23 genes: *ATM, BARD1, BRCA1, BRCA2, BRIP1, CDH, CHEK2, EPCAM, MLH1, MRE11A, MSH2, MSH6, MUTYH, NFN, NF1, PALB2, PMS2, PTEN, RAD50, RAD51C, RAD51D, STK11,* and *TP53.* Patient 5 underwent a more comprehensive gene panel test due to her significant personal and family history of cancer that included 49 genes: *APC, ATM, BAP1, BARD1, BRCA1, BRCA2, BRIP1, BMPR1A, CDH1, CDK4, CDKN2A, CHEK2, EPCAM FH, FLCN, GREM1, MAX, MEN1, MET, MITF, MLH1, MRE11A, MSH2, MSH6, MUTYH, NBN, NF1, PALB2, PMS2, POLD1, POLE, PTEN, RAD50, RAD51C, RAD51D, RET, SDHA, SDHAF2, SDHB, SDHC, SDHD, SMAD4, SMARCA4, STK11, TMEM127, TP53, TSC1, TSC2,* and *VHL.* Patient 3 underwent a gene panel specific to breast cancer risk that included 14 genes: *ATM, BARD1, BRIP1, CDH1, CHEK2, MRE11A, MUTYH, NBN, PALB2, PTEN, RAD50, RAD51C, STK11,* and *TP53*. Patient 10 underwent Lynch syndrome testing (*MLH1, MSH2,* and *MSH6*) in addition to *BRCA1/2* due to a personal history of early onset endometrial cancer. No pathogenic variants were identified in the other genes tested.

### DNA isolation

Hematoxylin and eosin-stained tissue 4 μm sections cut adjacent to unstained sections were examined by a pathologist. Using the stained slide as a guide, approximately 2mm^2^ of tumor tissue was manually scraped from the unstained slides. The tissue was digested in a solution of 1.0 mg/mL proteinase K (Sigma) in10mM Tris, pH 8.3, and 50 mM KCl. Digestions proceeded overnight at 56 °C. After fluorometric confirmation of adequate DNA concentration, the lysate was used directly for sequencing analysis.

### Library preparation

The Nimblegen Cancer Gene panel was used in this study. Sequences were selected using biotinylated capture probes (Nimblegen, Hoffmann-La Roche, Ltd., Basel, Switzerland). The captured DNA was fragmented to an average DNA fragment size is 180–220 bp and end-repaired for ligation of adaptor sequences carrying primer binding sites. The DNA was then amplified with primers tailed with sequencing primer binding sites and patient-specific indexes.

### Tumor DNA sequencing

That captured library (576 target genes) was sequenced on the MiSeq™ system (Illumina). The products of the indexing amplification were denatured and introduced to the flow cell for in situ amplification by bridge PCR on the MiSeq. The resulting clusters of immobilized templates were subjected to reversible dye terminator sequencing.

### Variant calling

Raw reads were aligned to human reference genome hg19 using BWA MEM [23]. Apparent PCR duplicates were removed using Picard Mark Duplicates [40]. Variants were called using FreeBayes variant detection [21] annotated for effect on protein sequence, predicted function, and frequency in different populations from the 1000 genomes project, and presence in variant databases COSMIC and ClinVar using Annovar [39].

## Results

### Variants

The eleven patients studied carried diagnoses of infiltrating ductal carcinoma, ductal carcinoma in situ, invasive lobular carcinoma, ovarian adenocarcinoma and primary peritoneal carcinoma. One patient with endometrial cancer (non-HBOC) was included. Patient ages at diagnosis ranged from 26 to 66 years. None of the 11 patient tumors tested displayed the familial *BRCA* variant (Table 1). All tumors underwent expanded gene panel testing and were confirmed negative for the familial variants.

**Table 1:** Variants with potential clinical significance. Putative somatic variants are identified as those with < 1% allele frequency in the 1000 genomes population and alternate allele frequency > 70% or < 30%, or those annotated in COSMIC

Case # Variant in First Degree Relative	Gene^a,b^	Loc	Variant	Read Depth	Variant Frequency^c^	Effect on Protein^d^	Population Frequency^e^
1 *BRCA2* c.C5645A	*PDE4DIP*	chr1	c.4993_4995CG	1084	0.39	NA	NA
	*FOXP1*	chr3	c.C343G:p.P115A	487	0.26	0.996	0.0005
	*PIK3CA* ^a^	chr3	c.A3140G:p.H1047R	654	0.28	0.639	NA
	*APC*	chr5	c.G7450A:p.G2484S	274	0.43	0.234	0.01
	*SMO*	chr7	c.C1939T:p.P647S	65	0.72	1	0.0032
	*CNTRL*	chr9	c.G5809A:p.E1937K	349	0.44	0.841	0.0018
	*TET1*	chr10	c.C767T:p.A256V	414	0.58	0.022	0.1
	*ZMYM2*	chr13	c.G454C:p.D152H	441	0.28	0.997	0.0023
	*ZMYM5*	chr13	c.A691G:p.T231A	105	0.21	0.014	0.09
	*MTUS2*	chr13	c.C2870T:p.T957 M	461	0.27	0.062	0.02
2 *BRCA1*c.IVS17 + 3 A > G	*EPHA10*	chr1	c.C205T:p.R69C	521	0.68	1	0.0009
	*ETV5*	chr3	c.C287T:p.S96F	3463	0.52	0.999	NA
	*CSF1R* ^a^	chr5	c.G1237A:p.G413S	2295	0.28	0.972	0.01
	*ROS1*	chr6	c.C3326T:p.S1109 L	3660	0.07	0.014	0.05
	*NACA*	chr12	c.A445G:p.K149E	2927	0.38	0.999	0.07
	*ZNF668*	chr16	c.G31A:p.D11N	3067	0.38	.	NA
	*BRCA1* ^a^	chr17	c.A926G:p.Q309R	2105	0.06	0.999	0.03
	*TP53* ^a^	chr17	c.A241G:p.K81E	1475	0.11	1	NA
3 *BRCA2* c.G8107A	*ERCC3*	chr2	c.C2111T:p.S704 L	59	0.29	0.642	0.0018
	*TET2*	chr4	c.C86G:p.P29R	62	0.25	0.993	0.09
	*PALLD*	chr4	c.A127T:p.T43S	142	0.62	0.992	0.0005
	*PDGFRB*	chr5	c.C1223G:p.S408C	51	0.33	1	0.0005
	*MLLT4*	chr6	c.A1264G:p.I422V	29	0.24	0.963	0.01
	*ROS1*	chr6	c.A1611G:p.I537M	61	0.44	0.275	0.07
	*KIAA1549*	chr7	c.G2800A:p.D934N	30	0.47	0.89	0.03
	*PTPRD* ^a^	chr9	c.C2983T:p.R995C	44	0.44	0.321	0.05
	*NUP98*	chr11	c.C3424G:p.Q1142E	38	0.54	0.359	0.06
	*TRIP11*	chr14	c.G2134A:p.E712K	37	0.27	0.979	0.01
	*MC1R* ^b^	chr16	c.G178 T:p.V60 L	34	0.32	0.988	0.05
4 *BRCA1* c.G5215A	*MYOC*	chr1	c.G227A:p.R76K	1834	0.37	0.984	0.08
	*ROS1*	chr6	c.G500A:p.R167Q	2239	0.46	0.908	0.06
	*FGFR1OP* ^a^	chr6	c.T760C:p.S254P	1276	0.66	0.007	0.03
	*FGFR1OP* ^a^	chr6	c.C470T:p.T157I	2039	0.68	0.868	0.09
	*FGFR1OP* ^a^	chr6	c.G672 T:p.K224 N	1821	0.70	0.001	0.09
	*KIAA1549*	chr7	c.G2800A:p.D934N	1214	0.47	0.89	0.03
	*TRIM24*	chr7	c.C2514G:p.D838E	3339	0.46	0.743	0.0027
	*RET* ^b^	chr10	c.C1946T:p.S649 L	1054	0.44	1	0.0009
	*NUP98*	chr11	c.C3424G:p.Q1142E	2139	0.54	0.359	0.06
	*HNF1A*	chr12	c.C293T:p.A98V	280	0.32	0.752	0.02
	*MC1R* ^b^	chr16	c.G178 T:p.V60 L	700	0.32	0.988	0.05
5 *BRCA1* c.A2841T	*MUC1*	chr1	c.G586A:p.G196S	2460	0.45	0.669	0.01
	*PAX3* ^b^	chr2	c.C941A:p.T314K	3586	0.42	0.837	0.0041
	*PAX8*	chr2	c.T985C:p.F329 L	2362	0.47	0.924	0.01
	*HECW1*	chr7	c.A3692G:p.N1231S	4508	0.46	0.998	0.0014
	*WRN*	chr8	c.G340A:p.V114I	3761	0.54	0.002	0.06
	*PTPRD* ^a^	chr9	c.C2983T:p.R995C	3365	0.44	0.321	0.07
	*NUP98*	chr11	c.C3424G:p.Q1142E	3553	0.54	0.359	0.06
	*CASC5*	chr15	c.A4339G:p.T1447A	4457	0.40	0.336	0.05
	*IGF1R* ^b^	chr15	c.G1532A:p.R511Q	3795	0.43	0.476	0.0009
	*CBFA2T3*	chr16	c.G308C:p.R103P	2797	0.43	0.811	0.04
	*SPECC1*	chr17	c.C579G:p.S193R	2353	0.60	1	0.1
	*SSX1*	chrX	c.A149G:p.Y50C	7574	0.46	0.97	0.0006
6 *BRCA1* c.187delAG	*FCRL4*	chr1	c.C34T:p.P12S	49	0.28	0.941	0.0046
	*MYOC*	chr1	c.G227A:p.R76K	24	0.37	0.984	0.01
	*KIAA1549*	chr7	c.G2800A:p.D934N	35	0.47	0.89	0.03
	*LTBP3*	chr11	c.C1313T:p.A438V	24	0.25	0.997	0.05
	*NUP98*	chr11	c.C3424G:p.Q1142E	27	0.54	0.359	0.06
	*BRCA2* ^b^	chr13	c.A865C:p.N289H	103	0.20	0.991	0.06
	*BRCA2* ^b^	chr13	c.A2971G:p.N991D	50	0.18	0	0.06
	*ERCC4*	chr16	c.G1244A:p.R415Q	52	0.48	1	0.03
	*MC1R* ^b^	chr16	c.G178 T:p.V60 L	17	0.32	0.988	0.05
	*CHD6*	chr20	c.C7165T:p.R2389C	29	0.53	1	0.006
7 *BRCA1* c.187delAG	*PDE4DIP*	chr1	c.A6002G:p.E2001G	927	0.58	0.999	0.1
	*PPARG* ^b^	chr3	c.C34G:p.P12A	923	0.46	0	0.07
	*ROS1*	chr6	c.G500A:p.R167Q	968	0.46	0.908	0.06
	*ROS1*	chr6	c.A1611G:p.I537M	1094	0.44	0.275	0.07
	*PMS2* ^b^	chr7	c.G59A:p.R20Q	877	0.45	0.924	0.07
	*HNF1A*	chr12	c.C293T:p.A98V	182	0.32	0.752	0.02
	*CBFA2T3*	chr16	c.G308C:p.R103P	676	0.43	0.811	0.04
*8* *BRCA1* c.3109insAA	*PDE4DIP*	chr1	c.A6002G:p.E2001G	42	0.58	0.999	0.1
	*FGFR1OP* ^a^	chr6	c.C470T:p.T157I	29	0.69	0.868	0.03
	*FGFR1OP* ^a^	chr6	c.T760C:p.S254P	22	0.66	0.007	0.03
	*ROS1*	chr6	c.G500A:p.R167Q	29	0.46	0.908	0.06
	*RET* ^b^	chr10	c.C1946T:p.S649 L	13	0.44	1	0.0009
	*NUP98*	chr11	c.G4759A:p.E1587K	25	0.79	1	0.01
	*NUP98*	chr11	c.C3424G:p.Q1142E	23	0.54	0.359	0.06
	*BLM*	chr15	c.C2603T:p.P868L	64	0.2	0.81	0.05
9 *BRCA1* c.1793 delA	*FANCD2*	chr3	c.A1634G:p.N545S	1102	0.27	0.06	0.0046
	*FGFR1OP*	chr6	c.G688C:p.A230P	1640	0.38	0.934	0.02
	*ROS1*	chr6	c.A1611G:p.I537M	1497	0.44	0.275	0.07
	*AKAP9*	chr7	c.G4519C:p.D1507H	1623	0.45	0.874	0.0005
	*MYC*	chr8	c.A77G:p.N26S	838	0.56	0.977	0.02
	*WRN*	chr8	c.C3236T:p.S1079 L	1327	0.29	0.405	0.02
	*CARS*	chr11	c.G38A:p.R13H	626	0.79	1	0.0032
	*NUP98*	chr11	c.G4759A:p.E1587K	952	0.79	1	0.01
	*BRCA2* ^b^	chr13	c.A865C:p.N289H	1907	0.20	0.991	0.06
	*BRCA2* ^b^	chr13	c.A2971G:p.N991D	1331	0.18	0	0.06
	*TSC2* ^b^	chr16	c.G1100A:p.R367Q	557	0.58	0.999	0.02
	*ASXL1*	chr20	c.C3692T:p.S1231F	1399	0.55	0.03	0.02
	*CHD6*	chr20	c.C7165T:p.R2389C	1628	0.53	1	0.01
10 *BRCA2* c.6794 insA	*ROS1*	chr6	c.G500A:p.R167Q	22	0.46	0.908	0.06
	*NUP98*	chr11	c.C3424G:p.Q1142E	10	0.54	0.359	0.06
	*OMD*	chr9	c.G662A:p.S221 N	41	0.46	0	0.03
	*ARNT*	chr1	c.T1506G:p.D502E	18	0.44	0.018	0.01
	*PAX3*	chr2	c.C941A:p.T314K	14	0.42	0.837	0.02
	*ARHGEF12*	chr11	c.A2861T:p.Y954F	68	0.33	0.01	0.05
	*PALB2*	chr16	c.G2993A:p.G998E	71	0.27	1	0.01
	*EZH2*	chr7	c.G436C:p.D146H	76	0.66	0.898	0.07
11 *BRCA2* c.6174 del T	*PAX3* ^b^	chr2	c.C941A:p.T314K	109	0.42	0.837	0.02
	*PPARG* ^b^	chr3	c.C34G:p.P12A	49	0.46	0	0.07
	*PMS2* ^b^	chr7	c.G59A:p.R20Q	76	0.45	0.924	0.07
	*MC1R*	chr16	c.G178 T:p.V60 L	34	0.32	0.988	0.05
	*MYOC*	chr1	c.G227A:p.R76K	84	0.37	0.984	0.08
	*PTPRD* ^a^	chr9	c.C2983T:p.R995C	44	0.44	0.321	0.07
	*SSX1*	chrX	c.A149G:p.Y50C	192	0.46	0.97	0.0006
	*MUC1*	chr1	c.G586A:p.G196S	110	0.45	0.669	0.01
	*FANCD2*	chr3	c.G521A:p.R174Q	34	0.21	0.96	0
	*CNTRL*	chr9	c.C6221A:p.A2074D	34	0.83	0.8	0.0041
	*PDE4DIP*	chr1	c.G2838A:p.M946I	25	0.59	0.028	0.09
	*PDE4DIP*	chr1	c.G4111A:p.V1371I	78	0.24	0.012	0.07

^a^Variant annotated in the Catalogue of Somatic Mutations in Cancer (COSMIC)

^b^Variant annotated ClinVar (NCBI Clinical Variant Database)

^c^Variant frequencies of 0.5 ± 0.05 or 1.0 ± 0.05 may be germline

^d^Polyphen algorithm score for predicting damaging mutations (non-Mendelian) (1 = most severe; http://genetics.bwh.harvard.edu/pph2/)

^e^Frequency reported in the combined 1000 genomes database

Between 2000 and 12,000 variants were detected per sample using the Nimblegen cancer gene panel. This panel is directed at high coverage of 572 genes with a reported role in carcinogenesis. Table 1 shows the analysis data for variants with potential clinical significance.

Variant data was annotated and screened for coverage (read depth or number of times sequenced). Samples 3, 6, 8, 10 and 11 had limiting DNA, resulting in lower coverage. The effect of the variant change on the protein product was calculated using PolyPhen for variants located in coding regions. The impact of the amino acid variants on protein structure (and function) was predicted from analysis of multiple sequence alignments and protein 3D-structures, predicting the effect of the DNA change on the cell (tumor) phenotype. All samples had at least one variant previously reported in the COSMIC or ClinVar databases. There are, however, caveats to using databases where submitted variants are at most classified into levels of clinical relevance as a source of information. Defined evidence categories provided by contributors may not sufficiently describe the medical significance of a variant [3].

The most frequently observed mutated gene was *ROS1* (6/11 cases), displaying variants p.S1109 L and p.I537M, neither of which predict a deleterious effect on protein function, and p.S1109 L, which may affect protein function (Polyphen score 0.908). *ROS1* encodes a receptor tyrosine kinase related to anaplastic lymphoma kinase (*ALK*), along with members of the insulin-receptor family. *ROS1* gene rearrangements lead to fusion of the entire tyrosine kinase domain of *ROS1* with 1 of 12 different partners, including fusions with: *TPM3, SDC4, SLC34A2, CD74* and *EZR*. *ROS1*gene rearrangements are present in about 1% of lung cancers where they are therapeutic targets for an FDA-approved agent Crizotinib. The same deleterious *RET* mutation p.S649 L (Polyphen score 1) was present in cases 8 and 4. *RET* is another receptor tyrosine kinase [10].

The next most frequent variant found was *NUP98*, found in 5/11 cases. This gene encodes a 186 kDa precursor protein that undergoes auto-proteolytic cleavage to generate a 98 kDa nucleoporin and 96 kDa nucleoporin, the latter portion is a scaffold component of the nuclear core complex that regulates transport of macromolecules between the nucleus and cytoplasm. Translocations between this gene and many other partner genes have been observed in myeloid leukemia and myelodysplastic syndrome [41].

Somatic *BRCA2* variants were observed in three tumors, two of which were from *BRCA1* phenocopies, and each had two *BRCA2* variants: N289H and N991D, only one of which is deleterious (Polyphen scores 0.991 and 0, respectively). A third tumor from a *BRCA1* phenocopy had a *BRCA1* Q309R deleterious variant (Polyphen score .999). All *BRCA* variants have been reported in COSMIC or ClinVar. None of familial *BRCA* pathogenic variants were found among the variants.

A *PALB2* p.E672Q variant (Polyphen score 0.275) was present in one tumor and a deleterious *PALB2* p.G998E variant found in another (Polyphen score 1). Both tumors were from *BRCA2* phenocopies. *PALB2* serves as the molecular scaffold in the formation of the homologous recombination *BRCA1*-*PALB2*-*BRCA2* complex [37].

Fanconi anemia complementation group D2, *FANCD2* variants p.N545S and p.R174Q were present in separate samples, the latter variant having predicted effects on protein function (Polyphen score .96), but also having low sequence depth. *PALB2* and *BRCA2* are members of this complementation group.

Other DNA repair complexes are represented amongst the variants including *PMS2* with p.V738F and p.R20Q, *ERCC3* p.S704 L and *ERCC4* p.R415Q. These variants, however, had low sequence coverage (< 50).

A somatic p.S1079 L variant in *WRN* was found in one case. Germline *WRN* variants are associated with premature aging. The *WRN* gene also functions in DNA repair and may have implications in tumorigenesis [9, 31].

Variants in genes that regulate histone and DNA methylation were present in three cases. *TET1* p.A256V has unlikely protein effect, while *TET2* p.P29R has a high Polyphen score .993. The ten-eleven translocation (*TET*) genes encode oxidases that demethylate methylated cytosine in DNA. A histone methylase gene variant, *EZH2* p.D146H (Polyphen score .898) was found in a different specimen. *EZH2* encodes a member of the Polycomb-group (PcG) protein family. PcG family members form multimeric protein complexes which maintain the transcriptional repressive state of genes.

Variants in genes that regulate histone and DNA methylation were present in three cases. *TET1* p.A256V is unlikely to affect the protein function, while *TET2* p.P29R has a high Polyphen score 0.993. The ten-eleven translocation (*TET*) genes encode oxidases that demethylate methylated cytosine in DNA. A histone methylase gene variant, *EZH2* p.D146H (Polyphen score .898) was found in a different specimen. *EZH2* encodes a member of the Polycomb-group (PcG) protein family. PcG family members form multimeric protein complexes which maintain the transcriptional repressive state of genes.

## Discussion

The current study addresses the origin of a disease phenocopy, an HBOC syndrome, in the absence of a familial (germline) gene pathogenic variant. We previously tested DNA from 11 tumors from women who come from families carrying *BRCA1* or *BRCA2* pathogenic variants, but who do not carry the variant themselves as defined by blood testing [28]. Although genetic alterations in any of the 11 tumor samples have not been demonstrated, several potential driver mutations were observed through testing with the Agilent 572 oncogene panel. There were no HBOC-related gene variants shared in all cases, although other somatic variants were present including *BRCA* variants in three cases and a *PALB2* variant in one case. The familial pathogenic variants in 6/11 cases are frameshift insertions and deletions and one intronic variant, while most of the detected pathogenic variants were exonic single nucleotide variant (SNV). This may be due to the gene panel used which is designed to detect SNV in exonic regions.

In the absence of available normal tissue from most of these cases, the identification of germline variants was limited. Based on allele frequencies highly divergent from 50% and low frequency in population studies of germline variation, some of the reported variants might be somatic. Some apparently benign variants such as *BRCA2* p.N991D and *PPARG* p.P12A are reported in ClinVar. ClinVar hosts germline and somatic variants. It contains all categories of germline variants including pathogenic, likely pathogenic, variants of uncertain significance (VUS), likely benign, and benign. ClinVar has 18 reports of *BRCA2* p.N991D, including familial breast and breast-ovarian cancer, Fanconi anemia, and HBOC syndrome. A germline mutation *PPARG* p.P12A was reported as a risk factor for obesity, noninsulin-dependent diabetes mellitus, and familial partial lipodystrophy was reported in a single study. Both of these variants were included in Table 1 because of their presence in the annotated database.

There was limited DNA for four of the 11 specimens, which is reflected in the coverage levels in Table 1. As a result of low coverage, the variants may not be included in a clinical report. For variants with strong clinical significance based on clinical data (called Tier 1 variants) [22], repeat sequencing or confirmation by other methods would be recommended. The rarity of *BRCA* phenocopies also limits this study. Additional samples would further confirm the lack of familial pathogenic variants as well as any commonalities in family variants or in the somatic variants of the phenocopies. Interestingly, the one patient with non-HBOC phenotype (endometrial carcinoma) had a complex family history of maternal HBOC history (breast and ovarian cancers) with pathogenic 6794 insA variant in *BRCA2* and paternal Lynch syndrome history (colon, lung and bone cancers) without any familial variant reported [28]. The possibility exists, therefore, that this patient is a phenocopy of a paternally inherited variant.

In addition to genetic drivers, epigenetic changes may also contribute to the disruption of cell phenotype and tumorigenesis. DNA hypermethylation in gene promoters is a mechanism for loss of function of genes. Epigenetic mechanisms independent of DNA sequence can affect phenotype in a heritable manner. DNA methylation patterns are reprogrammed during gamete production by erasure of epigenetic tags (DNA methylation and histone alterations). This should reset the DNA imprinting but rare survival of imprinted genes may persist [18, 29]. Furthermore, loss of function epigenetic regulators such as *TET* and *EZH2* through DNA mutation, may result in aberrant methylation in offspring.

The presence of variants that can alter methylation state led to the investigation of *BRCA 1/2* gene promoter methylation in the phenocopy DNA as measured by cytosine methylation. Aberrant DNA methylation patterns in gene promoters are strong regulators of gene expression and phenotype. In a preliminary analysis, *BRCA* promoter methylation status in tumor tissue DNA from phenocopies was compared to the DNA methylation in tumor tissue of *BRCA* carriers. Results so far in a small number of samples show a threefold increase in *BRCA*2 promoter methylation in phenocopy tumor tissue compared to tumor tissue from *BRCA* PV carriers suggesting that *BRCA2* promoter methylation in the phenocopy tumor tissues (from familial *BRCA*2 backgrounds) is consistently higher in phenocopy tumor DNA than in non-malignant and tumor tissue from the control germline *BRCA* PV carriers. A more thorough analysis of the *BRCA* promoter regions in tumor and matched non-malignant tissue will confirm the presence of the increased methylation and studies of directed methylation in cell lines would demonstrate the actual effect.

A complex cancer phenotype is probably not driven solely by a single genetic or epigenetic variant, even in the presence of a highly penetrant familial pathogenic variant [5, 30, 38]. The standard polygenic model of carcinogenesis proposes phenotypes produced by multiple loci acting independently and additively [13, 26]. The contribution of multiple loci could explain observed genetic inheritance characteristics, such as phenotypic variability, penetrance and anticipation. A combination of single nucleotide polymorphisms (SNPs), which singularly may not produce a malignant phenotype, may establish a genetic context within which PVs in high-penetrance cancer genes (or other variants not classified as such) can produce it [7, 15]. In the current study, *ROS*1 PVs were repeatedly observed. Either the genetic background promotes *ROS1* to a driver mutation, or *ROS1* is a passenger to a yet undiscovered driver mutation in another gene. Theoretically, then, a somatic or germline *BRCA* variant may be a driver only in the context of particular combinations of other variants. This idea is consistent with results of an exploratory study by Agarwal et al. which identified cancer gene germline-somatic mutation pairs that co-occurred more frequently than would be expected by chance. The authors concluded that germline polymorphisms might function as pre-existing driver “hits”, which together with acquired complementary somatic mutations would act to dysregulate key pathways in malignant transformation [1].

A familial genetic context would provide both conditions, with inheritance of a familial *BRCA* PV being manifested when present. In the absence of germline *BRCA* PVs, a combination of variants in other genes (or *BRCA* promoter methylation) may take this role. The polygenic inheritance might also affect the penetrance of the driver mutation through generations, as observed with anticipation phenomenon where cancer develops at a younger age in subsequent generations in some *BRCA*-positive families [19]. Once established, the polygenic background may select for additional variants or variant losses which increase the “malignant context,” i.e. establish an environment with greater cancer risk.

## Conclusions

An initial hypothesis that *BRCA* phenocopies were secondary to chimerism was not confirmed in our previous study. This prompted further analysis by extensive sequencing of DNA derived from tumor cells, to look for further insight into the pathogenesis of these tumors. The sequencing results confirmed that none of the familial pathogenic variants were present in the tumors of *BRCA* phenocopies. It also revealed several presumably somatic variants with potential oncologic significance. At least one variant in each case was previously reported in annotated databases. Somatic mutations in *ROS1* were the most frequently represented in this small case group, but their significance in breast and ovarian cancer is unknown at this time. Several presumably somatic variants were found in DNA repair genes which share homologous DNA repair function with *BRCA1* and *BRCA2* and are in the same Fanconi anemia pathway. Epigenetic silencing through increased DNA methylation and/or polygenic background context can be other underlying mechanisms explaining *BRCA* phenocopies.

## Data Availability

Deidentified complete sequence data analyzed during the current study are available from the corresponding author on reasonable request.
